# Preparation of Nanocomposites of Poly(ε-caprolactone) and Multi-Walled Carbon Nanotubes by Ultrasound Micro-Molding. Influence of Nanotubes on Melting and Crystallization

**DOI:** 10.3390/polym9080322

**Published:** 2017-07-30

**Authors:** Cristian Olmo, Hegoi Amestoy, Maria Teresa Casas, Juan Carlos Martínez, Lourdes Franco, Jose-Ramon Sarasua, Jordi Puiggalí

**Affiliations:** 1 Departament d’Enginyeria Química, EEBE, Universitat Politècnica de Catalunya, Edifici I.2, C/ Eduard Maristany, 10-14, Barcelona 08019, Spain; olmocristian@gmail.com (C.O.); m.teresa.casasl@upc.edu (M.T.C.); lourdes.franco@upc.edu (L.F.); 2 Departament of Mining Metallurgy Engineering and Materials Science, University of the Basque Country (UPV/EHU), Bilbao 48013, Spain; hegoy.amestoy@ehu.es (H.A.); jr.sarasua@ehu.eus (J.R.S.); 3 ALBA Synchrotron Light Facility, Ctra. BP 1413 km. 3,3, Cerdanyola del Vallès, Barcelona 08290, Spain; guilmar@cell.es; 4 Research Center for Multiscale Science and Engineering, Universitat Politècnica de Catalunya, C/ Eduard Maristany, 10-14, Barcelona 08019, Spain

**Keywords:** micro-molding technology, ultrasounds, nanocomposites, carbon nanotubes, crystallization, morphological parameters, synchrotron radiation

## Abstract

Ultrasound micro-molding technology was successfully applied to prepare nanocomposites based on a poly(ε-caprolactone) (PCL) matrix and multi-walled carbon nanotubes (MWCNTs). Optimization of processing parameters (i.e., amplitude, force and time) was crucial to obtain nanocomposites without any evidence of degradation, high material saving and short processing time (7–8 s). Good dispersion of nanotubes was achieved after processing previously formed solvent casting films. This dispersion was even partially detected in pieces directly obtained from powder mixtures of both components. Incorporation of MWCNTs had a remarkable influence on melting and crystallization processes, which were systematically studied by time resolved synchrotron experiments. Results indicated higher melting and crystallization temperatures for the nanocomposite, with temperature differences higher than 5 °C. Carbon nanotubes were effective nucleating agents and had an influence on crystallinity, crystallization rate and even on lamellar morphology, which was evaluated by analysis of the correlation function of small angle diffraction profiles. Crystallinity within lamellar stacks was lower for the solvent casting nanocomposite, but in this case lamellae underwent a thickening process during heating that accounted for the increase in the melting temperature. Crystallization from the melt rendered similar lamellar morphologies at the end of the process due to a lamellar insertion mechanism.

## 1. Introduction

**Figure 1:**
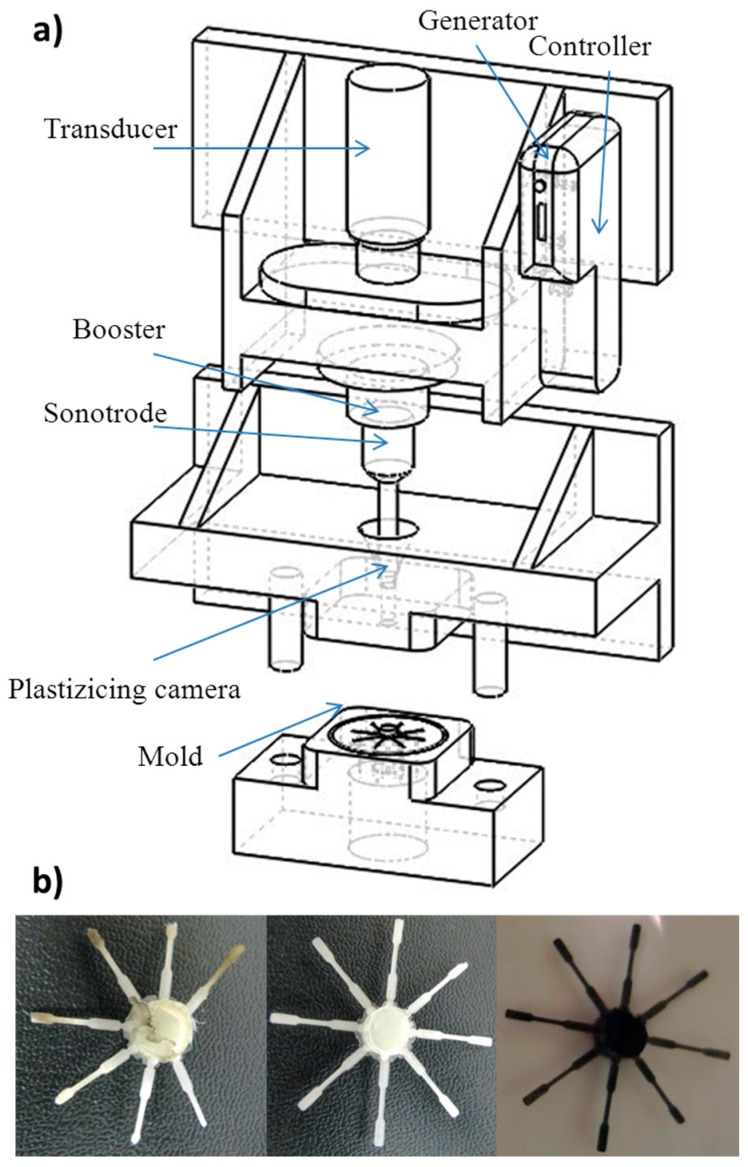
(**a**) Scheme of the main parts of the ultrasound micro-molding machine; (**b**) micro-molded pieces of PCL (left and middle) and PCL/MWCNT-5 (left) specimens processed under optimized (middle, right) and non-optimized (left) processing conditions.

The use of an ultrasonic source has been proposed as an alternative and effective processing method to prepare micro-molded pieces. The process may offer advantages over conventional micro-injection techniques and even may be highly interesting to obtain nanocomposites with good dispersion of nanoparticles [1,2,3]. The technology allows plasticization of the material and direct injection of the molten material [4,5,6]. Micro-molding equipment (Figure 1) is simple and requires only a plasticizing chamber, a controller, an ultrasonic generator to produce high frequency (30 kHz) from line voltage, a resonance stack or acoustic unit connected to the generator and a mold. The main element is the acoustic unit, which consists of: (a) a converter (piezoelectric transducer), where high frequency signals from the generator are transmitted through piezoelectric crystals that expand and contract at the same rate as electrical oscillation; (b) a booster, which amplifies or reduces mechanical oscillation (from 0 to 137.5 μm); and (c) a sonotrode, which transfers this oscillation to the polymer sample placed in the plasticizing chamber by applying a force (from 0 to 3500 N).

Ultrasonic waves have physical effects on the melt rheology of the polymer, facilitating the injection process, but also have chemical effects that could lead to polymer chain degradation. This is the result of cavitation and the possible high temperature inside the plasticizing chamber [7,8,9,10]. Therefore, processing parameters must be optimized in order to obtain minimally degraded samples. It was demonstrated that polylactide can be easily micro-molded [11], and so can other polyesters such as poly(butylene succinate) [1] and poly(nonamethylene azelate) [3], although in these cases the processing window was narrower.

Ultrasound technology was recently proven to be particularly interesting due to its ability to render exfoliated clay nanocomposites even when inorganic polar clays were mixed with apolar polymers (e.g., polylactide) [1]. This feature is significant since more expensive organo-modified clays must usually be employed to improve compatibilization with the organic polymer matrix when conventional processing techniques are applied (e.g., solvent casting, melt mixing or in situ polymerization). Furthermore, it should be considered that the use of an organo-modifier compound can have a negative effect on the thermal stability of the final nanocomposite because it may enhance thermal decomposition. That being said, typical nanocomposites should have better properties (e.g., barrier, physical, chemical, and mechanical properties) than those attained with conventionally filled composites [12,13,14]. 

Nanocomposites incorporating multi-walled carbon nanotubes (MWCNTs) are being widely studied because good conductive properties can be achieved, in addition to the typical advantages derived from the high surface to volume ratio of the reinforcing phase. It is, however, well-known that this kind of nanocomposites has serious problems concerning the high agglomeration trend of carbon nanotubes.

Poly(ε-caprolactone) (PCL) is a degradable and biocompatible polymer that is being widely employed as an implantable biomaterial. The main limitations of PCL are a consequence of its relatively poor mechanical and thermal properties, which justifies the recent intensive work on the preparation of composites with different nanofillers [15,16].

The present study has two main goals: (a) The evaluation of the suitability of ultrasound technology to produce micro-molded nanocomposites of MWCNTs and poly(ε-caprolactone) as a polymer matrix, insisting on their ability to keep adequate dispersion of nanotubes and an unaltered polymer molecular weight after processing, and (b) The evaluation of the influence of MWCNTs on the melting and crystallization processes as well as on the derived morphology. 

## 2. Materials and Methods

### 2.1. Materials

Poly(ε-caprolactone) was purchased from Solvay (Warrington, UK). The number average molecular weight was 128,900 g/mol and the polydispersity index 2.2, as determined by GPC. Graphistrength^®^ C10 carbon nanotubes were purchased from Arkema (Lyon, France), with their main characteristics being 10–15 nm diameter, 1–10 µm length, 90% purity and 5–15 walls.

### 2.2. Preparation of Nanocomposites

5 mg of PCL was dissolved in 50 mL of chloroform while 0.25 mg of MWCNTs was dispersed in 2.5 mL of chloroform by sonication for 20 min at 40% of maximum energy. Subsequently, the solution and dispersion were mixed while ultrasounds (Sonopuls HD2200, Bandelin, Berlin, Germany) were applied for another 10 min at 60% of maximum energy. The solvent was then evaporated in a Petri dish and the final film dried under vacuum in an oven at 120 °C. Thus, the nanocomposite contained 5 wt % of MWCNTs. It will hereafter be named PCL/MWCNT-5.

### 2.3. Micro-Molding Equipment

A first-generation prototype Ultrasound Molding Machine (Sonorus®, Ultrasion S.L., Barcelona, Spain) was employed. The apparatus was equipped with a digital ultrasound generator from Mecasonic (1000 W–30 kHz, Barcelona, Spain), a controller (3010 DG digital system, Mecasonic, Barcelona, Spain), a converter, an acoustic unit, and an electric servomotor control (Berneker and Rainer, Barcelona, Spain) fitted with software from Ultrasion S.L. The mold was thermally controlled and designed to prepare eight test specimens of dimensions 1.5 cm × 0.1 cm × 0.1 cm and complying with IRAM-IAS-U500-102/3 standards.

### 2.4. Measurements

Molecular weight was estimated by gel permeation chromatography (GPC) using a liquid chromatograph (Shimadzu, model LC-8A, Tokyo, Japan) equipped with an Empower computer program (Waters, Massachusetts, MA, USA). A PL HFIP gel column (Polymer Lab, Agilent Technologies Deutschland GmbH, Böblingen, Germany) and a refractive index detector (Shimadzu RID-10A, Tokyo, Japan) were employed. The polymer was dissolved and eluted in 1,1,1,3,3,3-hexafluoroisopropanol containing CF_3_COONa (0.05 M). The flow rate was 1 mL/min, the injected volume 20 μL, and the sample concentration 2 mg/mL. Polymethyl methacrylate standards were used to determine the number and weight average molecular weights and molar-mass dispersity.

Calorimetric data were recorded by differential scanning calorimetry using a TA instrument Q100 series equipped with a refrigerated cooling system operating from −50 to 300 °C. Experiments were performed under a flow of dry nitrogen with a sample of ∼5 mg. The instrument was calibrated for temperature and heat of fusion using an indium standard. Tzero technology required two calibrations, with empty pans and sapphires discs. Thermal characterization was carried out following a three run protocol consisting in a heating run (20 °C/min) of the initial sample, a cooling run (10 °C/min) after keeping the sample in the melt for 3 min and a second heating run (20 °C/min) of the nonisothermally crystallized sample.

Homogeneous thin films were successfully obtained by melting 1 mg of the studied sample on microscope slides. Small sections of these films were pressed between two cover slides which were subsequently placed on a hot stage. Samples were kept for 3 min at a temperature 10 °C higher than the melting point to wipe out sample history effects and then quickly cooled to the selected crystallization temperature. Nucleation was determined by optical microscopy using a Zeiss Axioscop 40 Pol light (Carl Zeiss, Göttingen, Germany) polarizing microscope equipped with a Linkam temperature control system configured by a THMS 600 heating and cooling stage connected to an LNP 94 liquid nitrogen vacuum pump and TP94 controller (Linkam Scientific, Tadworth, UK). Micrographs were taken with a Zeiss AxiosCam MRC5 digital camera (Carl Zeiss, Göttingen, Germany). A first-order red tint plate was employed to determine the sign of spherulite birefringence under crossed linear polarizers.

Distribution of MWCNTs in the composites was evaluated by means of a Philips TECNAI 10 electron microscope (Philips Electron Optics, Eindhoven, Holland, The Netherlands) at an accelerating voltage of 100 kV. A Sorvall Porter-Blum microtome (Sorwall, New York, NY, USA) equipped with a diamond knife was used to cut the sample in thin sections that were subsequently collected in a trough filled with water and lifted onto carbon-coated copper grids.

A Focused Ion Beam Zeiss Neon40 microscope (Zeiss, Oberkochen, Germany) operating at 5 kV was used to obtain SEM micrographs of micro-molded specimens. Carbon coating was accomplished with a Mitec K950 Sputter Coater (Quorum Technologies Ltd., Ashford, UK). 

Real-time synchrotron studies at variable temperature were performed confining the polymer samples between Kapton films and then placing them on a Linkam hot stage with temperature control within ±0.1 °C. Diffraction profiles were acquired during heating and cooling runs in time frames of 10 s and rates of 5 °C/min. 

Combined SAXS (small angle X-ray scattering) and WAXD (wide angle X-ray diffraction) experiments were carried out in the Non Crystalline Diffraction beamline, BL-11, at ALBA synchrotron light source (www.albasynchrotron.es). Polymer samples were confined between Kapton films and then placed on a Linkam hot stage with temperature control within ± 0.1 °C. Diffraction profiles were acquired during heating and cooling runs in time frames of 10 s and rates of 5 °C/min. 

The energy of the incident photons was 12.4 keV or equivalently a wavelength, λ, of 0.1 nm. The SAXS diffraction patterns were collected by means of a photon counting detector ImXPAD S1400 (ImXPAD, La Ciotat, France) with an active area of 152 × 149.6 mm^2^, an effective pixel size of 130 × 130 µm^2^ and a dynamic range of 32 bits. The sample-to-detector distance was set to 6163 mm, resulting in a *q* range with a maximum value of *q* = 2 nm^−1^. 

Additionally, the WAXS diffraction patterns were collected by means of a 3 CCD detector Rayonix LX255-HS (Rayonix LLC, Evanston, IL, USA) with an active area of 85 × 255 mm^2^, an effective pixel size of 44 × 44 µm^2^ and a dynamic range of 16 bits. In this case, the sample-to-detector distance was set to 225 mm, corresponding to a maximum *q* value of 62 nm^−1^. This detector was tilted with a pitch of 29.8°.

The data reduction was treated by pyFAI python code (ESRF), modified by ALBA beamline staff, that is able to do on-line azimuthal integrations from a previously calibrated file. The calibration files were created from well-known standards, i.e., silver behenate (AgBh) and Cr_2_O_3_ for SAXS and WAXS respectively. 

WAXD peaks were deconvoluted with the PeakFit v4 program by Jandel Scientific Software (V 4.0). The correlation function and corresponding parameters were calculated with the CORFUNC program for Fiber Diffraction/Non-Crystalline Diffraction provided by Collaborative Computational Project 13 (CCP13) (Chester, UK).

Characteristic lamellar parameters (i.e., long period, *L_γ_*, amorphous layer thickness, la, and crystalline lamellar thickness, *l_c_*) and crystallinity (i.e., crystallinity within lamellar stacks, *X_c_^SAXS^* = *l_c_*/*L_γ_*) were determined by the normalized one-dimensional correlation function [17], *γ*(*r*):

*γ*(*r*)=∫^∞^_0_*q*^2^*I*(*q*)cos(*qr*)d*q*/∫^∞^_0_*q*^2^*I*(*q*)d*q*, (1)

where *I*(*q*) is the intensity at each value of the scattering vector (*q* = 2π/*d*, where *d* is the Bragg spacing).

SAXS data were collected within a limited angular range, with application of the Vonk’s model [18] and Porod’s law to perform extrapolations to low and high *q* values.

## 3. Results and Discussion

### 3.1. Ultrasound Micro-Molding of PCL and PCL/MWCNT Nanocomposites

**Figure 2:**
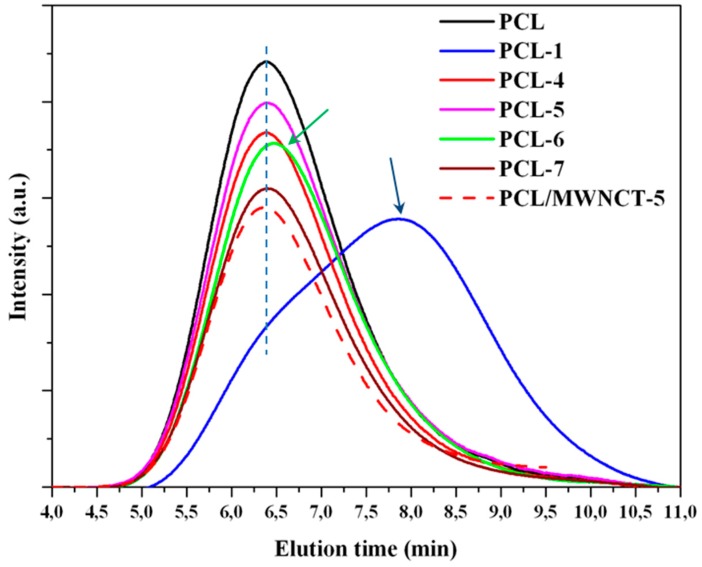
GPC molecular weight distribution curves determined for selected micro-molded PCL and PCL/MWCNT-6 specimens.

Processing parameters (i.e., amplitude, force and time) were optimized to obtain appropriate melt rheology and minimum degradation of polymer chains. The first point was evaluated by optical inspection of the morphology of the specimens (i.e., sprue and eight test specimens obtained after processing, as shown in Figure 1b for some representative micro-molded pieces) and considering the ability of the polymer to completely fill the mold cavities. The second point was quantitatively determined through GPC measurements, as shown in Figure 2 and Table 1 for some selected cases.

The optimization process started considering a relatively high force of 2000–2500 N despite the fact that previous works indicated that degradation of polyesters such as polylactide could be significant when a high force/pressure was applied even if the energy/amplitude was kept at a low level [11]. It was specifically suggested that chain scissions were enhanced by high mechanical shear stresses generated under a high force which, however, facilitated melt flow and complete filling of the mold cavities. A low force was also demonstrated ineffective [11] since it was not enough to reduce the melt viscosity of the polymer. On the other hand, the applied ultrasonic oscillations led to slight or significant polymer degradation. This effect could be observed upon comparison of the molecular weight of samples PCL-2 and PCL-3 or PCL-5 and PCL-7 (Table 1). Data demonstrate a significant molecular weight decrease (i.e., from 56,700 g/mol to 47,000 g/mol and from 57,500 g/mol to 47,400 g/mol), although the force was minimally varied from 2500 N to 2000 N.

Amplitude/energy played a relevant role in the degradation process of PCL. It was thus determined that amplitude could not exceed a value of 43 µm. Note that the GPC curve of samples processed at this amplitude (sample PCL-1 in Figure 2) shows a shift of the peak to clearly higher retention times than those observed for the neat PCL sample. Nevertheless, a shoulder corresponding to the initial population of PCL chains could still be observed. Obviously, the generation of small polymer fragments led to a high decrease of the number average molecular weight (i.e., from 58,100 g/mol to 5900 g/mol) and an increase of the polydispersity index (from 2.2. to 8.4), as summarized in Table 1. 

Optimization of processing time was also fundamental despite the short time required to perform ultrasound micro-molding. Note that this, together with the high material saving associated with this process (i.e., the efficient dosage precision allows the large amount of material usually rejected in conventional processing techniques to be reduced [4]), is probably the greatest advantage of the micro-molding technique over conventional micro-injection. In this way, assays performed for 9 s and keeping optimal amplitude and force (sample PCL-6 in Table 1) gave rise to processed specimens with a yellow/brown coloration (Figure 1b) indicative of degradation, a slight shift of the GPC curve (Figure 2) and a significant decrease of the molecular weight (*M*_n_ = 37,100 g/mol, Table 1). By contrast, short test times led to deficient filling of the mold (e.g., 6 s was insufficient, as indicated in Table 1 for samples PCL-2 and PCL-3). Times of 7 s and 8 s appeared ideal (Table 1); specifically, complete filling of the mold and a molecular weight identical to that determined for the neat polyester were obtained with amplitude and force values of 37 µm and 2500 N, respectively (sample 4 in Table 1 and Figure 2). These optimized conditions were also satisfactory for processing the nanocomposite (Table 1 and Figure 1b and Figure 2). In summary, the new ultrasound technology was suitable to produce micro-pieces with minimum degradation and high processing speed of both PCL and its nanocomposite with a large ratio of MWCNTs (i.e., up to 5 wt %). Furthermore, it was demonstrated [11] that micro-pieces with high precision details (e.g., around 50–100 µm) can be well molded. By contrast, conventional micro-injection techniques require the application of very high pressures [19].

**Figure 3:**
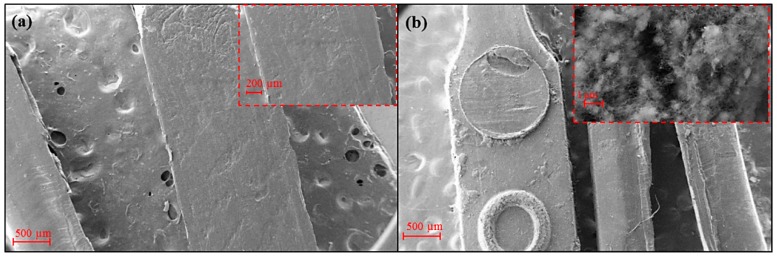
SEM micrographs showing details of micro-molded PCL (**a**) and PCL/MWCNT-5; (**b**) specimens. Magnifications are given in the insets.

The morphology of PCL and PCL/MWCNT samples processed under optimized conditions was observed by scanning electron microscopy (SEM). Figure 3a is a micrograph of a transverse section of a PCL specimen showing the high homogeneity of the material and lack of pores and cracks. Figure 3b shows the outer surface of the PCL/MWCNT-5 sample (see external marks used for numbering the pieces). A smooth and relatively homogeneous surface can be observed, although a few small pores may be detected. Note that loaded MWCNTs can be clearly observed in the enlarged image of a specific pore.

**Figure 4:**
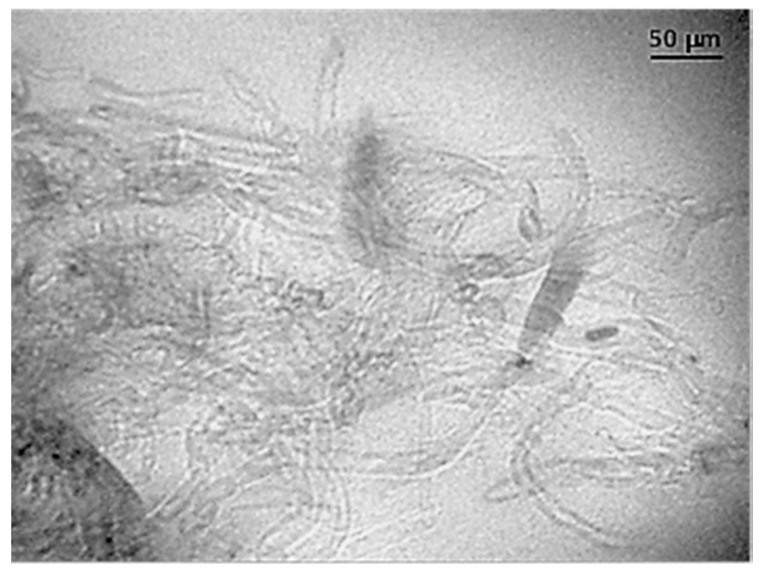
TEM micrograph showing dispersion of MWCNTs in the PCL nanocomposite incorporating 5 wt % of nanotubes.

In order to evaluate the ability of ultrasounds to disaggregate MWCNTs into powder and render homogeneous dispersion in the final micro-molded specimen, direct molding of PCL and MWCNT powder mixtures without using solvent casting nanocomposite films was assayed. Results were promising although more effort is required to optimize conditions. That being said, well-formed micro-pieces without evidence of degradation were obtained using the above processing parameters. TEM images reveal the existence of zones where MWCNTs were well dispersed inside the PCL matrix (Figure 4), but unfortunately some aggregates (not shown) were also detected, as expected from the inherent difficulty of dispersing nanotubes at loads as high as 5 wt %.

### 3.2. Thermal Properties of PCL/MWCNT Nanocomposites

**Figure 5:**
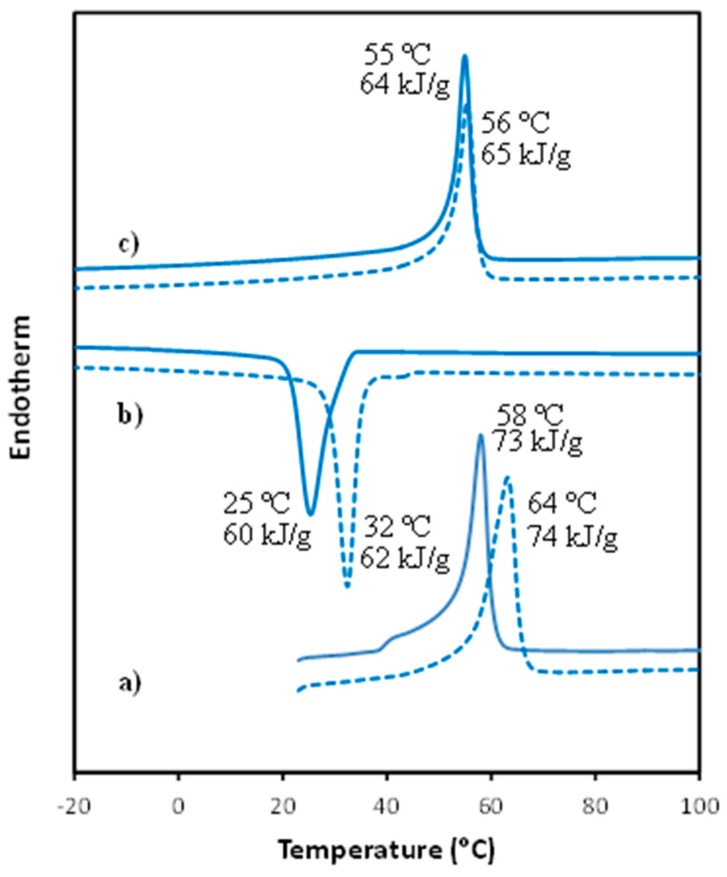
DSC scans performed with PCL (solid lines) and PCL/MWCNT-5 specimens (dashed lines): (**a**) Heating of solvent casting films; (**b**) Cooling from the melt state and (**c**) Heating of melt crystallized samples.

Incorporation of carbon nanotubes had a significant influence on both melting and crystallization processes, as shown in Figure 5, where the sequence of heating-cooling-reheating scans performed with PCL (solid lines) and PCL/MWCNT-5 (dashed lines) samples is displayed. Incorporation of nanotubes increased the melting and corresponding crystallization temperatures of solvent casting films. By contrast, the influence on the melting temperature of melt crystallized samples was low (1–2 °C), which may be indicative of different molecular arrangements of the nanocomposite that depend on the way it is prepared (i.e., by solvent casting or from the melt). 

It should also be pointed out that the crystallization peak is clearly narrower for the nanocomposite, indicating a faster crystallization process and suggesting an enhanced nucleation effect when nanotubes were incorporated. Finally, melting and crystallization enthalpies were rather similar for the two samples, with slightly higher values for the nanocomposite. Note that enthalpies of nanocomposites for the first heating, cooling and second heating runs were 79, 65 and 68 kJ/g if the 5 wt % content of MWCNT is considered.

### 3.3. Melting and Crystallization of PCL and PCL/MWCNT-5 Nanocomposites from Time-Resolved WAXD Experiments

**Figure 6:**
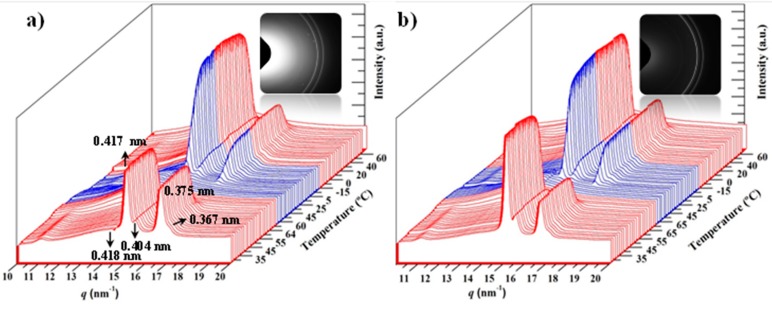
Three dimensional representations of WAXD profiles of PCL (**a**) and the PCL/MWCNT-5 nanocomposite; (**b**) during a sequence involving heating (red), cooling (blue) and subsequent heating (red) runs performed at a rate of 5 °C/min. Insets show the corresponding 2D diffraction patterns taken at room temperature.

In order to study in detail the influence of these nanoparticles in the melting and crystallization processes, time-resolved WAXD profiles of the nanocomposite having 5 wt % of MWCNTs were taken during a heating-cooling-reheating sequence (Figure 6). Diffraction profiles of the neat polyester were also taken for comparative purposes. 

The first heating run was performed with solvent casting samples to start with materials crystallized at the same temperature (i.e., room temperature) and avoid any effect attributable to differences in the initial morphology (e.g., variation in lamellar thickness) that could be expected if samples were non-isothermally processed from the melt state (e.g., by injection or micro-molding). 

X-ray diffraction profiles showed an amorphous halo centered at 0.418 nm and four diffraction peaks at 0.417, 0.404, 0.375 and 0.367 nm. These are indexed as (110), (111), (200) and (201) reflections of the rectangular unit cell, with *a* = 0.748 nm, *b* = 0.498 nm and *c* = 1.726 nm, as postulated for poly(ε-caprolactone) [20,21,22]. The X-ray diffraction profile of the initial PCL sample was peculiar since the intensities of (110) and (200) reflections were very similar, in contrast with the clear predominance of the (110) reflection in the profile of the nanocomposite (see also the 2D-diffraction patterns in the insets of Figure 6) as well as in the profiles of the PCL and nanocomposite obtained at 25 °C of samples cooled from the melt state (see cooling runs in Figure 6). Differences in the packing mode of PCL chains and even the molecular conformation (all trans and non-planar conformations have been proposed in the literature for PCL [21]) are thus observed depending on the preparation method and the incorporation of MWCNTs. In any event, a high intensity of the (200) reflection indicates that the average plane defined by the methylene carbon atoms of a molecular chain tends to be parallel to the ac crystallographic plane.

Significant differences were detected in the evolution of the WAXD profiles of the nanocomposite and the pristine polymer because the decrease in the intensity of the four Bragg peaks (i.e., the beginning of fusion) started at a higher temperature for the nanocomposite (i.e., 63 °C versus 52 °C). This suggests differences in the lamellar morphology and the associated recrystallization process, which are discussed in the next section.

**Figure 7:**
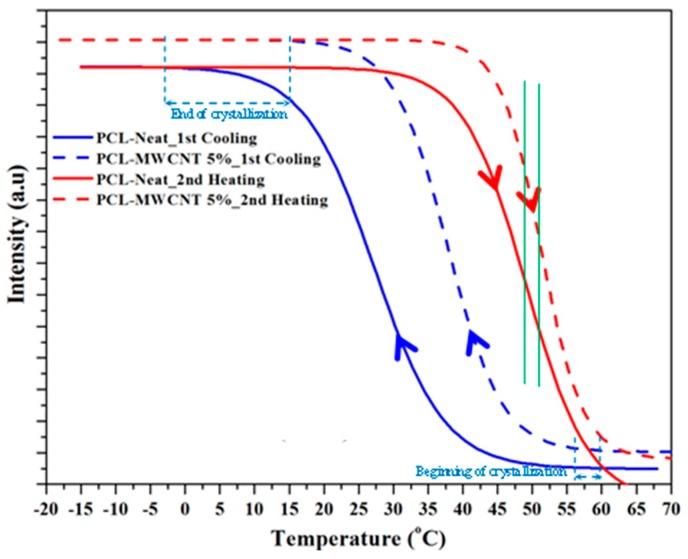
Variation in intensity of the main WAXD reflection during cooling (blue) and the subsequent heating (red) runs performed at a rate of 5 °C/min for PCL (solid lines) and the PCL/MWNCT-5 nanocomposite (dashed lines).

The subsequent cooling runs of the two samples were also very different because the crystallization of the nanocomposite started at a higher temperature (60 °C as opposed to 51 °C) and ended at a higher temperature too (15 °C as opposed to −3 °C). The plot of the intensity of the strongest peak versus temperature (Figure 7, blue lines) clearly shows the lower supercooling needed for the nanocomposite (see dashed double arrow) and the faster crystallization process, which could also be deduced from the higher slope of the linear representation.

**Figure 8:**
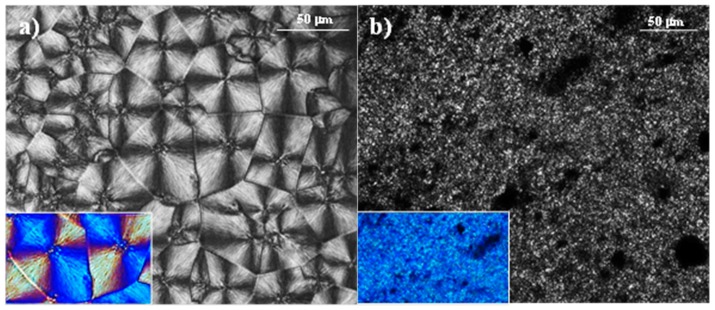
Polarized optical micrographs of PCL (**a**) and PCL/MWCNT (**b**) samples crystallized from the melt state at 48 °C.

This faster crystallization rate is a consequence of the nucleation induced by the nanoparticles, as also revealed by optical microscopy micrographs taken during isothermal crystallization at a given temperature. Specifically, Figure 8 shows that PCL crystallizes at 48 °C, giving rise to large negatively birefringent spherulites with a mean diameter of 50 µm and a low nucleation density (i.e., 800 nuclei/mm^2^). By contrast, MWCNTs prevented the growth of PCL in large size spherulites (Figure 8b) due to their strong nucleating effect. On the other hand, the nanocomposite sample was highly crystalline, as evidenced by its strong overall birefringence. The morphology of nanocomposites loaded with different MWCNT percentages was always similar even for a minimum load of 0.5 wt % (not shown). It has been reported that the high nucleation effect of MWCNTs can lead to peculiar morphologies; specifically, nanohybrid shish-kebab structures with CNTs as shish and polymer crystals as kebab have been reported for polyethylene and nylon 66 based nanocomposites [23]. 

The crystalline structures of PCL and the nanocomposite were similar when samples were crystallized from the melt. Therefore, minimum differences in the melting temperature should be expected, as shown in the DSC profiles recorded during the second heating run (Figure 5). Nevertheless, it is also highly interesting to point out that melting started at a significantly higher temperature for the nanocomposite (i.e., 37 °C versus 32 °C, see Figure 7). Finally, the plot of the evolution of the intensity of the main reflection during heating (Figure 7) also shows a higher slope for the nanocomposite, indicating a more homogeneous lamellar population that undergoes the melting process. In any event, the temperature corresponding to the maximum slope (see green lines in Figure 7) was similar (difference lower than 2 °C) for both samples, in agreement with the identical melting peak temperature observed in the DSC scan.

**Figure 9:**
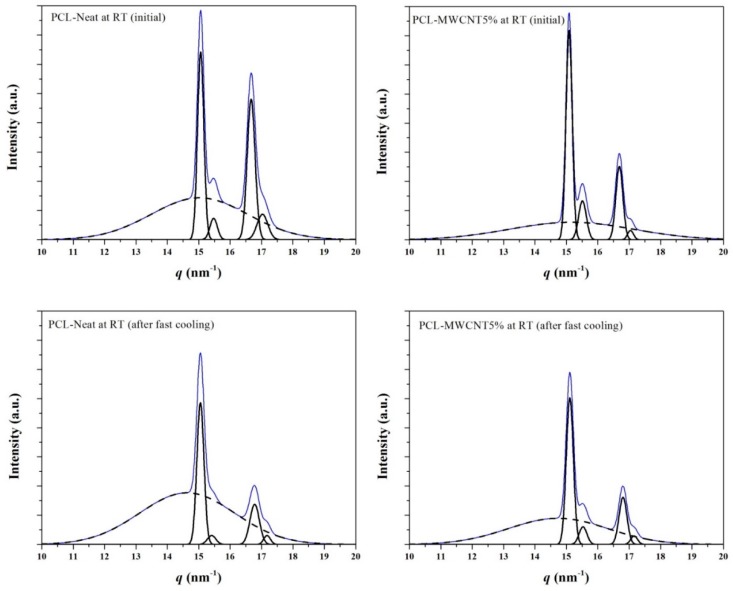
Deconvoluted X-ray diffraction profiles of PCL (**a**,**c**) and PCL/MWNCT-5 (**b**,**d**) samples taken at room temperature at the beginning of the heating process (**a**,**b**) and after being cooled from the melt state at the maximum rate allowed by the equipment (50 °C/min) (**c**,**d**).

Deconvolution of X-ray diffraction profiles (Figure 9) allowed sample crystallinity, XcWAXD, to be accurately estimated by measuring the intensity of Bragg reflections and the amorphous halo. Results indicate a higher degree of crystallinity for the nanocomposite probably as a consequence of enhanced nucleation, which even favors the development of solvent casting crystals. Thus, *X_c_^WAXD^* values of 40% and 50% were determined for the initial PCL and PCL/MWCNT-5 samples, respectively. A slight increase was found when samples crystallized from the melt but the higher value was still observed for the nanocomposite. Thus, crystallinities of 42% and 51% were determined at room temperature (before end of the crystallization process) for PCL and PCL/MWCNT-5 samples, respectively. These crystallinities increased to 47% and 53% at the end of the crystallization process. Figure 9 also shows the profiles obtained with melt-quenched samples, with the expected significant decrease of *X_c_^WAXD^*. Again, a higher value was determined for the nanocomposite (i.e., 38% versus 23%). 

Some contradictions are found between DSC melting enthalpies and the calculated *X_c_^WAXD^* values. They can be explained by the fact that the high melting enthalpies observed for the solvent casting films and specifically for the PCL sample are due to an enhanced hot crystallization process during heating and to an energetically more favorable molecular arrangement in the peculiar PCL structure. Otherwise, neither the above crystallinities of 40% and 50% nor the higher crystallinities determined for the melt crystallized samples can be justified. 

### 3.4. SAXS Analysis of the Melting Crystallization of PCL and PCL/MWCNT Nanocomposites 

**Figure 10:**
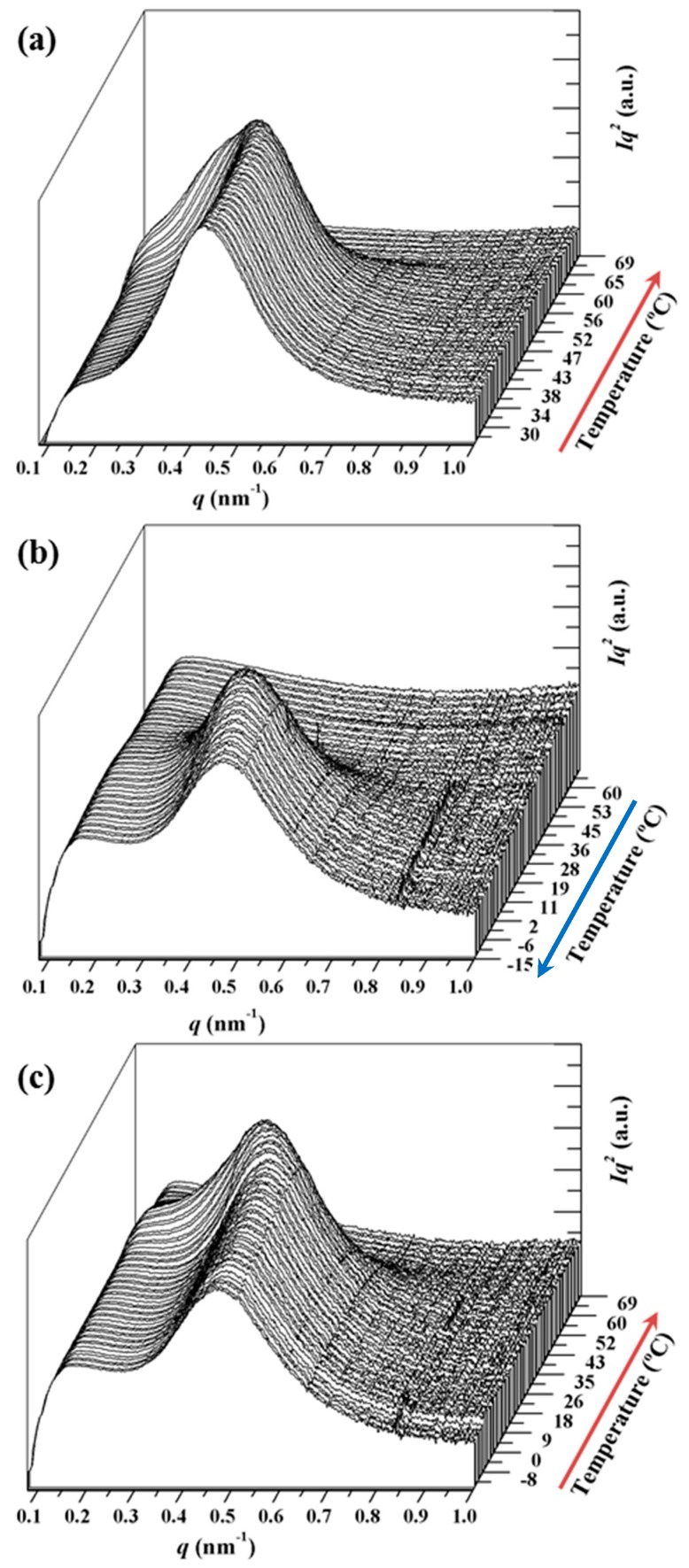
Three dimensional representations of PCL SAXS profiles taken during first heating (**a**), cooling; (**b**) and second heating and (**c**) runs performed at a rate of 5 °C/min.

**Figure 11:**
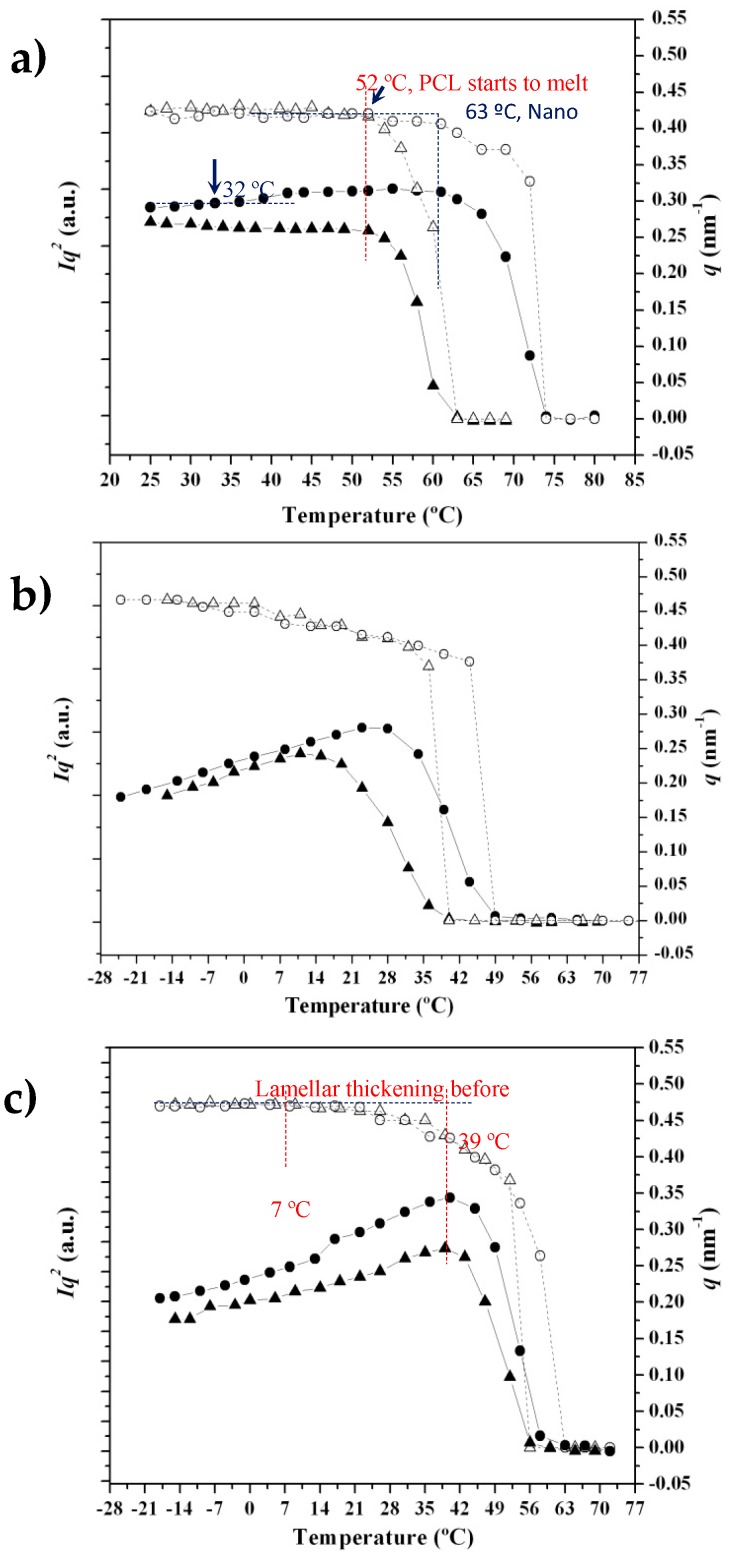
Variation in intensity (*Iq*^2^) (full symbols) and scattering vector (*q*) (empty symbols) of the SAXS peak observed in the diffraction profiles taken during the first heating run (5 °C/min) (**a**), the subsequent cooling run from the melt state (5 °C/min); (**b**) and the second heating run and (**c**) of PCL (triangles) and PCL-MWCNT-5 nanocomposite (circles).

Time-resolved SAXS profiles of the neat PCL sample taken during the heating-cooling-reheating sequence (Figure 10) are clearly different from those acquired for the nanocomposite sample. Analyses could be easily performed using the plots in Figure 11, which show the evolution of intensity (*I_q_*^2^) and the corresponding value of the *q* scattering vector of the SAXS peak during the heating and cooling processes for both samples. 

Figure 11a reveals differences between profiles taken during the first heating. PCL showed a practically constant value of intensity and *q* until the beginning of fusion at 52 °C, while a significant increase of intensity at temperatures higher than 32 °C and a slight decrease of *q* from 52 °C until the beginning of fusion (63 °C) was characteristic of the nanocomposite. The increase observed for the peak intensity can be associated with the higher contrast between the electron density of the crystalline and amorphous phases. It seems that a lamellar reorganization took place, leading to an increase in the lamellar thickness, as demonstrated by the decrease of the scattering vector, and in the chain mobility of the amorphous phase, resulting in a lower density and higher contrast. These morphological changes were very important above 52 °C (see red line in Figure 11a, which corresponds to the beginning of fusion for the neat PCL). Thus, the nanocomposite underwent recrystallization and expansion at the temperature at which the neat polymer melted. 

The evolution of intensity and *q* during cooling for both samples can be seen Figure 10b. The intensity data clearly show that the nanocomposite started to crystallize at a higher temperature (52 °C as opposed to 42 °C), leading to densification of the amorphous phase (as deduced from the decrease in peak intensity) at a higher temperature (28 °C as opposed to 12 °C). This compaction process was more evident for the nanocomposite. Both samples showed an increase of *q* at lower temperatures, which could be attributed to the decrease of the crystallization temperature. This results in thinner lamellae and a typical lamellar insertion mechanism once crystallization has proceeded extensively (e.g., temperatures lower than 28 and 12 °C associated with the maximum intensity). In this case, lamellae crystallized in the confined space between the previously formed lamellae. *q* values were logically practically constant at temperatures lower than 10 °C, coinciding with the end of the crystallization process. 

Figure 11c shows the evolution during the subsequent heating process. As can be seen, the most interesting characteristics were the intensity increase, which reached a maximum value at a similar temperature for both samples (i.e., 39 °C), a clear recrystallization process (lamellar thickening) that also occurred at a similar temperature for both samples (note the evolution of *q* between 7 and 39 °C) and a melting process that was also similar for both samples (i.e., melting temperature of 63 versus 61 °C). In this way, the influence of carbon nanotubes was different depending on the way the nanocomposites were obtained (i.e., solvent casting or melt crystallization). 

**Figure 12:**
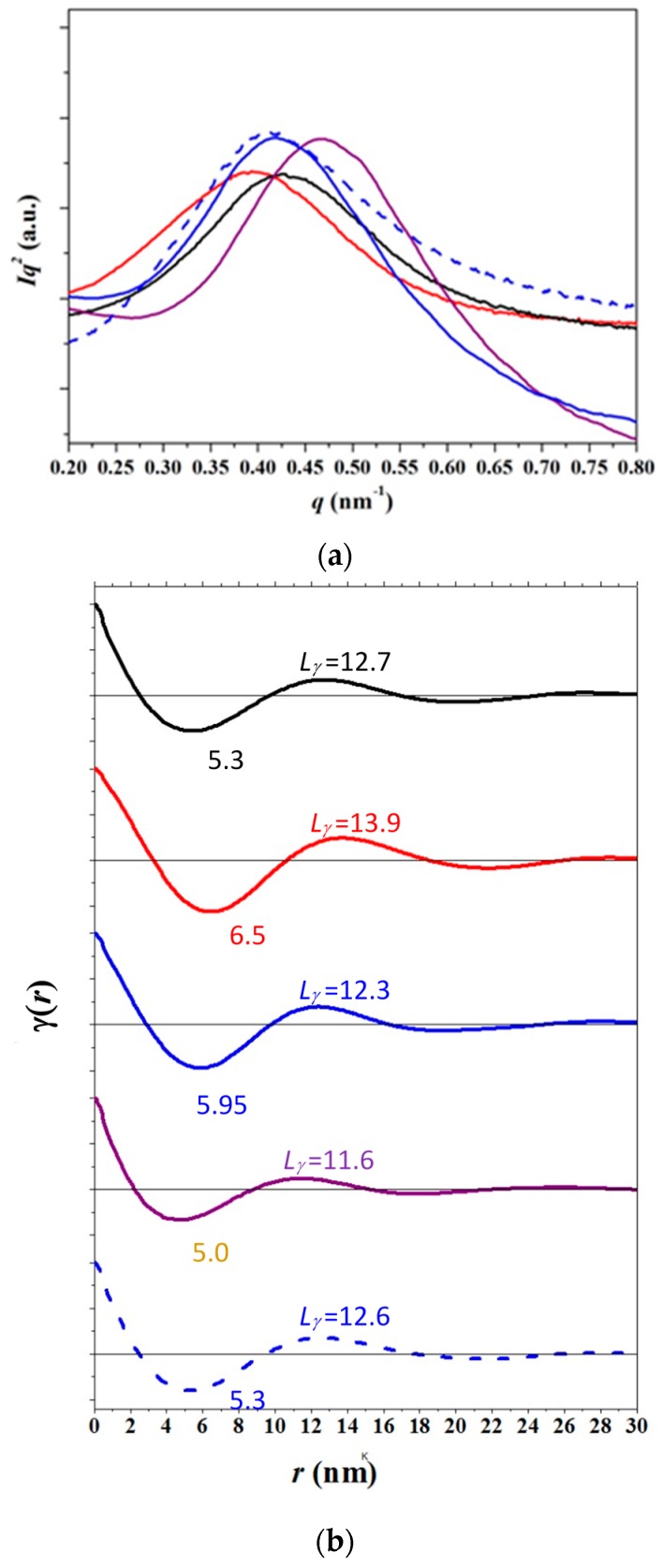
SAXS profiles (**a**) and corresponding correlation functions (**b**) for initial PCL (black), PCL heated to 55 °C (red), non-isothermally crystalized PCL at 25 °C (blue) and −10 °C (violet). For the sake of completeness, the correlation function of the non-isothermally melt crystallized nanocomposite at 25 °C (blue dashed line) is shown.

Morphological lamellar parameters were analyzed by the correlation function that corresponds to the Fourier transform of the SAXS profile. Representative profiles and the corresponding correlation functions are shown in Figure 12. It can be observed that profiles taken at room temperature of the initial sample (i.e., that obtained by solvent casting) and after melt crystallization are similar with respect to the value of the scattering vector associated with the peak but clearly different with respect to peak width (Figure 12a), which is indicative of a broader lamellar distribution.

In fact, discrepancies between the *L_γ_* value (derived from the first maximum of the correlation function) and the long period, *L_γ_*^m^ (determined by doubling the value of the first minimum of the correlation function) are informative about the distribution of the layer widths of the major component. This was assigned to crystalline layer thickness in a typical two-phase lamellar model although the correlation function cannot distinguish the thickness associated with each phase (i.e., amorphous and crystalline). This feature is explained below and has been well reported for other polymers [24,25,26]. 

Broader lamellar distributions are associated with *L_γ_* values clearly larger than *L_γ_^m^* long period [27]. The latter are representative of the most probable distance between the centers of gravity of a crystal and its adjacent amorphous layer whereas the former are associated with the most probable distance between the centers of gravity of two adjacent lamellar crystals. Obviously, the two parameters should be practically identical for a regular distribution, as observed at room temperature for the partially melt crystallized sample (i.e., 12.3 nm and 11.9 nm). By contrast, *L_γ_* and *L_γ_^m^* spacings of 12.7 nm and 10.6 nm, respectively, were determined for the more irregular solvent casting sample prepared. Interestingly, lamellar distribution broadened at the end of melt crystallization (i.e., at −15 °C) since different and lower spacings were found (i.e., 11.6 nm and 10 nm). This is a clear indication of an irregular distribution caused by the lamellar insertion process. Finally, the function determined at 55 °C supports again a relatively broad distribution caused, in this case, by the lamellar thickening process (i.e., *L_γ_* and *L_γ_*^m^ spacings increased to 13.9 nm and 13 nm, respectively). 

**Figure 13:**
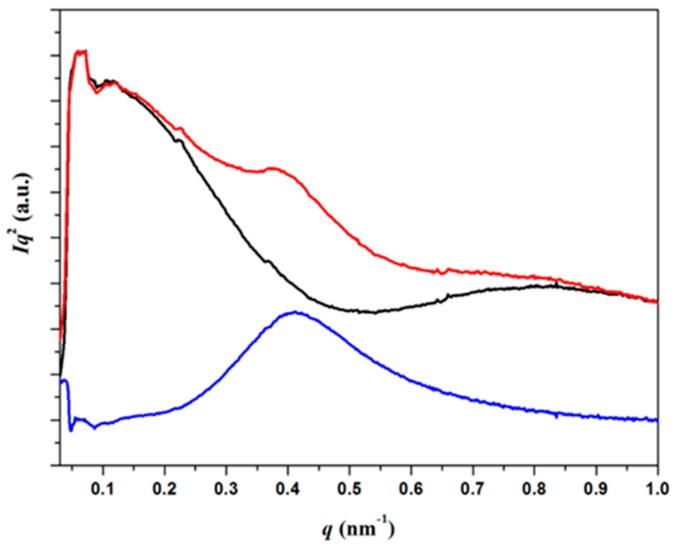
SAXS diffraction profiles of PCL/MWCNT-5 specimens taken at room temperature (red) and after fusion (black). The diffraction profile obtained after subtraction is indicated by the blue line.

Similar features were observed for the nanocomposite sample, although the analysis was more difficult because the scattering of MWCNTs had to be separated from the scattering contribution due to PCL crystallization (Figure 13) [28]. In general, lamellar distributions were more irregular. For example, *L_γ_* and *Lγ^m^* spacings of 12.6 nm and 10.3 nm were respectively determined for a PCL/MWCNT-5 melt crystallized sample by the correlation function at 25 °C (dashed line in Figure 12b). 

**Figure 14:**
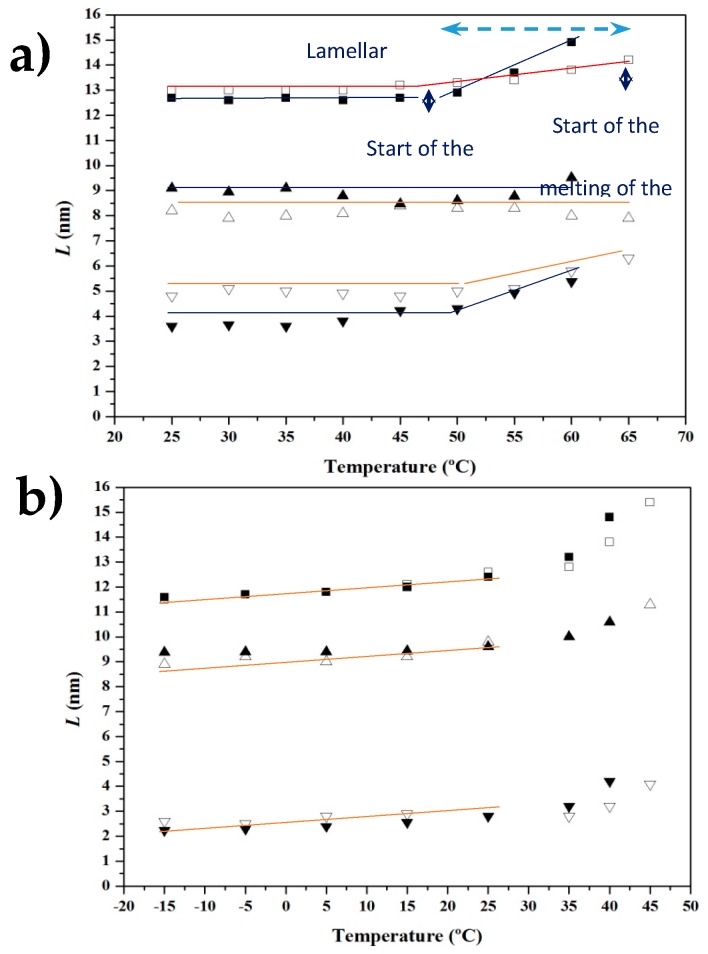
Plots showing the variation of the long spacing (■,□) and the crystalline (▲, Δ) and amorphous (▼, ▽) layer thicknesses of PCL (full symbols) and PCL/MWCNT-5 (empty symbols) during the first heating run (5 °C/min) (**a**) and the subsequent cooling run (5 °C/min) (**b**).

Analysis of the evolution of *L_γ_*, *l_c_* and l_a_ morphological parameters during heating and cooling scans for the neat PCL and the nanocomposite samples by the correlation function revealed significant differences between the two samples, as displayed in Figure 14a. Crystallinity within lamellar stacks, *X_c_^SAXS^*, was easily calculated as *l_c_*/*L_γ_* and allowed the thickness associated with each phase to be assigned. The derived crystallinities should always be greater than the crystallinity deduced from WAXD data (*X_c_^WAXD^*) because samples should always contain amorphous-rich domains that are not considered in the simple lamellar biphasic model associated with the correlation function analysis. Specifically, *X_c_^SAXS^* values of 64% and 72% were determined for the solvent casting samples of the nanocomposite and the neat PCL, respectively.

Basically, the three morphological parameters remained practically constant up to the temperature at which the neat PCL started to melt (i.e., 52 °C). At higher temperatures, the amorphous lamellar thickness of the neat PCL increased drastically as a consequence of the fusion, whereas a moderate change was observed for the nanocomposite because only a reordering process occurred in the temperature interval between 52 and 63 °C. Samples crystallized from solution according to slightly different morphologies. Specifically, the presence of MWCNTs led to crystals with greater and lower amorphous and crystalline layer thicknesses, respectively. Nanoparticles seem to have a nucleating effect, as previously indicated, but also influence the degree of perfection of lamellae. Probably, lamellae with the greatest amorphous contribution are more susceptible to undergoing reordering, leading to a higher melting point. Note also that the lower *X_c_^SAXS^* crystallinity determined for the nanocomposite is not in contradiction with its higher *X_c_^WAXD^* crystallinity because nucleation results in more but more imperfect crystals.

The evolution during cooling (Figure 14b) shows the initial formation of thicker lamellae for the nanocomposite because of the nucleation effect and the higher temperature at which crystallization started. Nevertheless, the subsequent evolution was similar for both samples, with similar values of *L_γ_*, *l_c_* and *l_a_* at low temperatures. This explains the similar behavior during the subsequent heating run. A similar *X_c_^SAXS^* crystallinity around 78% was determined for both samples, which was logically higher than that observed for solvent casting samples.

## 4. Conclusions

It was demonstrated that ultrasound micro-molding is a suitable technology to process poly(ε-caprolactone) as well as its nanocomposite with MWCNTs, even for high loads (e.g., 5 wt %). Amplitude, force and processing time need to be optimized in order to guarantee appropriate melt rheology to fill mold cavities completely and avoid molecular degradation. Thus, amplitude should be lower than 43 µm, time between 7–8 s and force close to 2500 N. Good dispersion of nanotubes was attained when solvent casting nanocomposite films prepared were processed. Even partial dispersion was obtained when a simple powder mixture of the two components was molded.

Incorporation of nanotubes had an influence on the melting and crystallization processes, as determined by DSC and X-ray diffraction data taken during real time heating and cooling experiments. Both melting and crystallization temperatures increased for the nanocomposite. In the latter case, a clear nucleating effect of nanotubes was derived. Crystallinity values of the solvent casting nanocomposite sample determined by WAXD were higher than those found by SAXS. The nanofiller enhanced crystallization due to its nucleating effect but also resulted in more imperfect crystals.

Morphological changes during heating were different for the neat polyester and the nanocomposite as a consequence of the different degree of perfection of lamellae. Specifically, amorphous layer thickness was greater for the nanocomposite, facilitating a reordering process that led to an increased melting point. The evolution of the morphological parameters was similar on cooling although the nanocomposite rendered thicker lamellae at the beginning of the crystallization process due to the nucleation effect and the higher crystallization temperature. 

## Figures and Tables

**Table 1:** Influence of micro-molding processing parameters on sample molecular weight.

Sample	Amplitude (µm)	Force (N)	Time (s)	*M*_n_ (g/mol)	*M*_w_ (g/mol)	PI
PCL	-	-	-	58,100	128,900	2.2
PCL-1 ^1^	43	2000	6	5900	49,200	8.4
PCL-2 ^1^	37	2000	6	47,000	120,100	2.6
PCL-3 ^1^	37	2500	6	56,700	127,300	2.2
PCL-4 ^2^	37	2500	8	58,100	128,900	2.2
PCL-5 ^2^	37	2500	7	57,500	128,100	2.2
PCL6 ^1^	37	2500	9	37,100	116,400	3.1
PCL-7 ^2^	37	2000	7	47,400	127,100	2.7
MWCNT-5 ^2^	37	2500	8	58,000	128,000	2.2

**^1^** The sample could not fill all mold cavities; ^2^ The sample filled all mold cavities.
